# Supplementation with Combined Additive Improved the Production of Dairy Cows and Their Offspring with Maintenance of Antioxidative Stability

**DOI:** 10.3390/antiox13060650

**Published:** 2024-05-27

**Authors:** Hongxing Zhang, Yiliyaer Nuermaimaiti, Kebi Hao, Yan Qi, Yiming Xu, Yimin Zhuang, Fei Wang, Guobin Hou, Tianyu Chen, Jianxin Xiao, Gang Guo, Yajing Wang, Shengli Li, Zhijun Cao, Shuai Liu

**Affiliations:** 1 State Key Laboratory of Animal Nutrition and Feeding, College of Animal Science and Technology, China Agricultural University, Beijing 100193, China; xingxing@cau.edu.cn (H.Z.); ylyer@cau.edu.cn (Y.N.); xuyiming1028@163.com (Y.X.); b20213040363@cau.edu.cn (Y.Z.); w15152786127@163.com (F.W.); guobin.hou@bua.edu.cn (G.H.); chentianyu@cau.edu.cn (T.C.); xiaojianxin-dairy@cau.edu.cn (J.X.); yajingwang@cau.edu.cn (Y.W.); lisheng0677@163.com (S.L.); caozhijun@cau.edu.cn (Z.C.); 2 Beijing Sunlon Livestock Development Co., Ltd., Beijing 100176, China; snhaokb@163.com (K.H.); guogang2180@126.com (G.G.); 3 China Animal Husbandry Group, Beijing 100070, China; qiy@cahg.com.cn; 4 College of Animal Science, Xinjiang Agricultural University, Urumqi 830052, China; 5 College of Animal Science and Technology, Beijing University of Agriculture, Beijing 102206, China

**Keywords:** transition cows, calf, additives, blood immunity, productive performance

## Abstract

Oxidative stress damage in periparturient cows decreases both production and their health; supplementation with complex additives during the periparturient period has been used as an important strategy to enhance the antioxidant status and production of dairy cows. The periparturient cows not only risk a negative energy balance due to reduced dry matter intake but also represent a sensitive period for oxidative stress. Therefore, we have developed an immunomodulatory and nutritional regulation combined additive (INC) that hopefully can improve the immune status and production of cows during the periparturient period and their offspring health and growth by improving their antioxidant stress status. The INC comprised a diverse array of additives, including water-soluble and fat-soluble vitamins, Selenomethionine, and active dry Saccharomyces cerevisiae. Forty-five multiparous Holstein cows were randomly assigned to three treatments: CON (no INC supplementation, *n* = 15), INC30 (30 g/d INC supplementation, *n* = 15), and INC60 (60 g/d INC supplementation, *n* = 15) based on last lactation milk yield, body condition score, and parity. Newborn calves were administered 4 L of maternal colostrum originating from the corresponding treatment and categorized based on the treatment received by their respective dams. The INC not only served to maintain the antioxidative stress system of dairy cows during the periparturient period but also showed a tendency to improve the immune response (lower tumor necrosis factor and interleukin-6) during the perinatal period. A linear decrease in concentrations of alkaline phosphatase postpartum and β-hydroxybutyrate was observed with INC supplementation. Milk fat yield, milk protein yield, and energy-corrected milk yield were also increased linearly with increasing additive supplementation. Calves in the INC30 group exhibited greater wither height and chest girth but no significant effect on average daily gain or body weight. The diarrhea frequency was linearly decreased with the incremental level of INC. Results indicate that supplementation with INC in peripartum dairy cows could be a major strategy to improve immune response, decrease inflammation, maintain antioxidant stress status in transition dairy cows, and have merit in their calves. In conclusion, this study underlines the benefits of INC supplementation during the transition period, as it improved anti-inflammatory capacity, could positively impact antioxidative stress capacity, and eventually enhanced the production performance of dairy cows and the health and growth of calves.

## 1. Introduction

The transition period, spanning three weeks before and after parturition, presents a complex challenge for dairy cows due to oxidative stress and immune and endocrine shifts that lead to physiological imbalances [1,2,3]. The increase in reactive oxygen species (ROS) during this period can lead to a number of diseases associated with oxidative stress, conditions that are associated with a reduced capacity of the antioxidant defense system [4,5]. High levels of ROS can lead to a dysfunctional immune response during the periparturient period and increase the risk of health disorders in cows and their calves [6]. Many studies have demonstrated that restricted energy intake or heightened oxidative stress in dairy cows during the transition period can significantly influence the immune and metabolic functions of their offspring [7,8,9]. Furthermore, these factors can adversely affect milk production and reproductive performance in the postnatal period [10], with potential repercussions extending into the first lactation of the offspring [11,12,13]. Human and mouse models have provided insights indicating that suboptimal intrauterine conditions during critical developmental periods can induce structural and functional alterations in fetal tissues [14]. A comprehensive review has indicated that oxidative damage can be mitigated or rectified through the utilization of novel feed additives, such as supplemented vitamins and micronutrients, which have demonstrated efficacy in improving the immune profile of animals [5]. As cows dry matter intake (DMI) decreases during the periparturient period, supplementation with antioxidants such as vitamins and micronutrients can minimize the effects of excess ROS, thereby improving animal health and reducing disease incidence [15]. Reduced concentrations of micronutrients and vitamins in the blood can impair the cow’s immune system during the periparturient period and lead to a variety of inflammatory diseases [16,17,18]. Supplementation with cellular antioxidants and immune system modulators such as vitamins (vitamin B12 and E) and minerals (selenium) significantly enhances the animal’s immune response and facilitates the rapid restoration of homeostasis [19,20,21]. Supplementation of organic selenium during the late periparturient period helped to improve antioxidant status and immune response after calving [22]. Supplementation with selenium and vitamin E during the transition period in dairy cows significantly reduced oxidative stress and lipid peroxidation, increased blood levels of antioxidant enzymes (glutathione and superoxide dismutase), and simultaneously improved udder immunity and health [23]. Moreover, a study has shown that supplementation with yeast culture also has positive impacts on milk production in dairy cows [24]. In addition, nutrient supplementation in periparturient cows affects the nutritional and immunological properties of the colostrum and ultimately the well-being of the newborn calves [25,26]. Previous studies have also demonstrated that the addition of rumen-protected B vitamins (choline, niacin, folic acid, etc.) positively impacts antioxidant capacity, pre-weaning immunity and growth performance in calves [27,28,29]. Therefore, we have developed an immunomodulatory and nutritional combined additive (INC) consisting of rumen-protected water-soluble and fat-soluble vitamins, Selenomethionine, and active dry Saccharomyces cerevisiae that may enhance the antioxidant capacity and immunity, improve energy metabolism, and ultimately improve the quality of colostrum in cows during the perinatal period, which may have more beneficial effects on offspring. In addition, most previous studies have only focused on the production performance of cows during the perinatal period [24,30,31]. However, the relationship between antioxidant and micronutrient supplementation during the periparturient period and cow immunity and calf performance was less known. Thus, the objective of this study was to investigate the effects of supplementing dairy cows with INC during the critical transition period, assessing the effects on antioxidative stress, immune, and hepatic functions, including evaluating the impact on productive performance, and examining the consequences for colostrum composition, as well as the growth and health of their offspring. Our hypothesis posited that the supplementation of INC during the transition period would yield improvements in antioxidative stress, immunity, productivity, and calf development.

## 2. Materials and Methods

### 2.1. Experimental Design and Animal Management

Forty-five multiparous Holstein dairy cows were blocked by the expected calving date, body condition scoring (BCS), parity, and 305-d milk yield of the last lactation (Table 1) and randomly assigned to 1 of 3 treatments: (1) 0 g/d INC, control group (CON; *n* = 15), (2) 30.0 g/d INC (INC30, *n* = 15), or (3) 60.0 g/d INC (INC60, *n* = 15). The INC consisted of Vitamin E (5.30%, rumen-protected), Vitamin K (0.07%, rumen-protected), Vitamin B (8.69%, rumen-protected), Selenomethionine (6.55%, rumen-unprotected), active dry yeast (19.9%, viable yeast ≥ 2 × 1010 CFU/g, rumen-unprotected), and limestone (59.5%). Treatments were top-dressed at each morning feeding between 06:30 and 07:00 h, beginning −21 d and continuing until 21 d after parturition. The average date before expected parturition for the 3 groups had no difference (22.1 vs. 21.9 vs. 22.5 d; *p* = 0.65) for CON, INC30, and INC60 when the experiment was started.

During the peripartum period, the cows were housed in the same freestall barn with no cubicles in order to provide more room for each cow (>10 m^2^ per cow). The barn was bedded with dried and fermented feces with rice hulls (20 cm deep). Fresh total mixed ration (TMR) was delivered at 06:30 and 14:00 h every day. All cows had free access to water via self-filling water troughs (3.0 × 0.5 m; length × width; Beijing Eastern Bell Technology Group, Beijing, China). The cows would be moved to a calving pen and bedded with straw as they neared calving, and fresh TMR and water would also be provided. After calving, the cows were moved to another freestall barn for freshcows equipped with cubics (1.85 m × 1.35 m, length to brisket locator × width), yielding a total stall length of 2.75 m. The cubicles were covered with rubber mats. The cows were fed TMR at 06:30, 14:00, and 20:00 h in the freshcow barn and had free access to water by self-filling water troughs (3.0 × 0.5 m; length × width; Eastern Bell Technology Group, Beijing, China). Cows were given adequate daily diets to ensure 3–5% orts. All the cows were milked three times after calving, at 06:00, 13:30, and 19:30 h, and the milk ALPRO monitoring system of DeLaval (Stockholm, Switzerland) automatically recorded the daily milk yield of each cow. The health status of each cow was checked and recorded by an experienced farm veterinarian and a member of the research team according to the standard operating procedure of the farm for the management of cow diseases. Abnormal cows were recorded and treated promptly with propylene glycol, glucose solution, antibiotics, etc. No cows experienced major symptoms that could affect the results during the whole experimental period.

### 2.2. Diets, Milk Production and Composition

Pre- and postpartum diets (Table 2) were formulated to meet the demand for net energy and crude protein in multiparous cows during the transition period (NASEM, 2021). We could not measure DMI in this study due to the trial being conducted on a commercial farm lacking the necessary equipment to track individual feed intake. Samples of TMR were collected once a week and stored at −20 °C for subsequent nutrient composition analysis. Each TMR sample was dried in a forced hot air drying machine at 60 °C for 48 h to determine dry matter (DM) and was ground through a 1-mm screen using a mill. The samples were then sent to the State Key Laboratory of Animal Nutrition and Feeding, China Agricultural University, to determine the DM, crude protein (CP), crude fat, and ash [32]. Neutral detergent fiber (NDF) and acid detergent fiber (ADF) content were determined according to the method provided by Van Soest [33]. The content of calcium and phosphorus in the feed was determined by atomic absorption spectrometry (ZA3000, Hitachi High-Tech Scientific Solutions Co., Ltd., Tokyo, Japan). The milk samples of 45 cows were collected on d 7, d 14, d 21 (06:00, 14:00, and 20:00 h for each sample day). After collection, the three milk samples of each cow from each sample day were mixed in a ratio of 4:3:3 with added preservatives and stored at 4 °C. The milk samples were tested within 72 h for determining milk protein, milk fat, lactose, urea nitrogen, and somatic cells using an automated near-infrared milk analyzer (Foss NIRS DS3, Foshwa Technology & Trade Co., Ltd., Hilleroed, Denmark) at the Beijing Dairy Center (Beijing, China).

### 2.3. Blood Sampling and Analyses for Dairy Cows

Blood samples were collected on d −21, d −7, d 0, d 7 and d 21 relative to calving before morning feeding via the coccygeal vein into a 10 mL evacuate tube without anticoagulant. Blood samples were immediately placed on ice and transported to the laboratory, followed by centrifugation at 3500× *g* for 15 min at 4 °C (LC-LX-HLR210D, Hunan Lichen Instrument Technology Co., Ltd., Changsha, China) to obtain serum. Serum samples are stored in 1.5-mL microcentrifuge tubes at −80 °C in a freezer until further analysis for insulin (INS), glucose (GLU), triglyceride (TG), β-hydroxybutyrate (BHB), NEFA, insulin-like growth factor-1 (IGF-1), glutamic oxaloacetic transaminase (AST), glutamic pyruvic transaminase (ALT), alkaline phosphatase (ALP), total bilirubin (TBIL), superoxide dismutase (SOD), total antioxidant capacity (T-AOC), glutathione peroxidase (GSH-Px), malondialdehyde (MDA), IgG, interleukin (IL)-1β, IL-6, tumor necrosis factor-α (TNF-α), serum amyloid A (SAA), and haptoglobin (HP). INS was measured by radioimmunoassay using a multitube radioimmunoassay counter (model BFM-96, Zhongcheng Electromechanical Technology Co., Ltd., Guangzhou, China). The concentrations of HP, IgG, SAA, IL-1β, IL-6, and TNF-α were measured with kits from Beijing Laibo Terui Technology Development Co., Ltd. (Beijing, China). The concentrations of AST, ALT, ALP, TBIL, SOD, T-AOC, GSH-Px, and MDA were determined using an automated biochemistry analyzer (model 7600, Hitachi High-Tech Scientific Solutions Co., Ltd., Kyoto, Japan). The NEFA, BHB, GLU, TG, and IGF-1 were measured using ELISA kits in an enzyme-immunoassay instrument (Multiskan MK3, ThermoFisher Scientific Co., Ltd., Waltham, MA, USA).

### 2.4. Colostrum Sampling and Analyses

After collection from dairy cows within 60 min, the colostrum was weighed, and part of the sample was collected and divided into two 15 mL centrifuge tubes at −20 °C for later composition analysis. One 15 mL sample was sent to the Beijing Dairy Center (Beijing, China) for analysis of the milk protein, milk lactose, and milk fat. Another 15 mL of colostrum was collected for the analysis of IgG, IgA, IgM, SOD, GSH-Px, T-AOC, catalase (CAT), and MDA concentrations via radial immunodiffusion assay. The concentrations of IgG, IgA, IgM, SOD, GSH-Px, CAT, and MDA in colostrum were determined using the double antibody sandwich method using an enzymatic calibrator (ReadMax 1200, Shanpu Biotechnology Crop., Shanghai, China) at 450 nm, and the T-AOC in serum was measured by ferric ion reducing antioxidant power using the same kits at 590 nm wavelength.

### 2.5. Calf Housing and Colostrum Management

All calves were born in the calving pen, separated from their dams within 30 min after birth, and weighed. Colostrum produced by cows after calving in the three groups was kept in the same environment. Calves were grouped by dam treatments in each group and fed the colostrum produced by the corresponding treatment group. Calves were then moved to a separate straw-bedded nursery room, where they were fed with 4 L of high-quality pasteurized colostrum (evaluated by a Brix refractometer, >22%) within 1 h after birth. After 24 h, the blood of calves was collected and examined for total protein. Only calves with successful passive transfer of immunity (serum total protein ≥ 5.2 g/dL) were included in the study and moved to individual hutches (1.5 m × 2.5 m; width × length). Each hutch was bedded with straw that was renewed weekly. From d 2 to d 56, calves were fed with pasteurized waste milk (nonsaleable whole milk) through a bucket (without teat). The nutritional composition of pasteurized waste milk and starter diets fed to calves is presented in Table 3. The feeding regime was as follows: 6 L per day from d 2 to d 7 (three times per day per calf), 7.5 L per day from d 8 to d 14 (three times per day per calf), 9 L per day from d 15 to d 42 (three times per day per calf), 7.5 L per day from d 43 to d 49 (three times per day per calf), 5 L per day from d 50 to d 52 (twice per day per calf), 4 L per day from d 53 to d 55 (twice per day per calf), and 2 L on d 56 (once per day per calf). All calves had free access to water, and pelleted starter was provided from d 3. After weaning on d 56, calves remained in their hutches with free access to the pelleted starter, oat hay, and water for 2 more weeks until the end of the study (d 70). All the feeding facilities were cleaned daily. To reduce environmental interference, all calves were raised in two adjacent rows of corrals, and all the calves were placed randomly in one of the hutches. The 45 calves born from the study were all successful in passive transfer of immunity and were blocked based on treatments of the dams.

### 2.6. Calf Growth Measurements, Feed Sampling, and Analysis

The body weight (BW) of the calf was measured on d 1, d 7, d 14, d 21, d 28, d 35, d 42, d 49, d 56, d 63, and d 70 before each morning milk feeding. After weighing the calves, body length (shoulders to pins), wither height, and heart girth were measured. Starter intake was recorded before each morning feeding based on the amount offered and refused by each calf. Starter samples (Table 3) were collected once per week and pooled for analysis of DM, CP, crude fat, and crude ash following the methods of AOAC (2006). NDF and ADF content were determined according to the method provided by Van Soest [33]. The enzyme-colorimetric method was used to determine the starch content of the starter [34]. Pasteurized waste milk samples were collected from d 1 to 56 once per week and sent to the Beijing Dairy Center (Beijing, China) for testing of their nutritional composition (Table 3).

### 2.7. Calf Health Check and Treatment

The health check of calves in this study included fecal scoring and a respiratory health check. The 4-point scoring was used for the diarrhea check, and the scores were 1 = firm, well-formed (not hard); 2 = soft, pudding-like; 3 = runny, pancake batter; and 4 = liquid, splatters, and pulpy orange juice [35]. Diarrhea was defined as any animal presenting a score of 3 or 4. The equation for calculating diarrhea frequency is as follows: diarrhea frequency (%) = [(number of diarrhea calves × days of diarrhea)/(total number of calves × days of trial)] × 100. Diarrhea calves were treated according to the protocols established by the farm veterinarian (e.g., by administering antibiotic drugs and electrolytic solutions). Respiratory health was checked after each morning feeding by visually inspecting nasal discharge and listening to breathing difficulties with auscultation by the farm veterinarian and a member of the research team. Throughout the study, no calves had respiratory disease.

### 2.8. Statistical Analysis

The MIXED PROC of SAS 9.4 (SAS Institute Inc., Cary, NC, USA) was used to calculate the data for this investigation, utilizing a randomized complete block design. Total morbidity in dairy cows was analyzed using a chi-square test. Blood metabolites, milk yield, and milk composition of dairy cows were analyzed as repeated measures according to the following model:

Y*_ijk_* = μ + B*_i_* + TRT*_j_* + W*_k_* + TRT × W*_jk_* + R*_n:ijk_* + e*_ijk_* (1)

where Y*_ijk_* = the dependent variable, μ = the overall mean, B*_i_* = the random effect of the *i*_th_ block, TRT*_j_* = the fixed effect of the *j_th_* treatment level, W*_k_* = the fixed effect of the kth week or day relative to calving, TRT × W*_jk_* = the fixed interaction between the *j*_th_ treatment level and the kth week, R*_n:ijk_* = the random effect of the nth animal and e*_ijk_* = the residual error term. The colostrum composition, including concentrations of IgG, IgA, IgM, SOD, T-AOC, GSH-Px, MDA, CAT, milk-fat, milk-protein, and milk-lactose of colostrum, was analyzed according to the following model:

Y*_ij_* = μ + B*_i_* + TRT*_j_* + R*_n:ijk_* + e*_ij_* (2)

where Y*_ij_* = the dependent variable, μ = the overall mean, B*_i_* = the random effect of the *i*_th_ block, TRT*_j_* = the fixed effect of the *j*_th_ treatment level, R*_n:ijk_* = the random effect of the nth animal, and e*_ij_* = the residual error term. 

Blood metabolites, growth measurements, and DMI of calves were analyzed as repeated measures according to the following model:

Y*_ijk_* = μ + B*_i_* + TRT*_j_* +W*_k_* + TRT × W*_jk_* + R*_n:ijk_* + e*_ijk_* (3)

where Y*_ijk_* = the dependent variable, μ = the overall mean, B*_i_* = the random effect of the *i_th_* block, TRT*_j_* = the fixed effect of the *j*_th_ treatment level of the dam, W*_k_* = the fixed effect of the week or day relative to calving, TRT × W*_jk_* = the fixed interaction between the *j*_th_ treatment level and the kth week, R*_n:ijk_* = the random effect of the nth animal and e*_ijk_* = the residual error term. Health data, including diarrhea rates and the frequency of calves, were analyzed using a chi-square test. Initial calf BW was analyzed according to the following model:

Y*_ij_* = μ + B*_i_* + TRT*_j_* + R*_n:ij_* + e*_ij_* (4)

where Y*_ij_* = the dependent variable, μ = the overall mean, B*_i_* = the random effect of the *i_th_* block, TRT*_j_* = the fixed effect of the *j_th_* treatment level of the dam, R*_n:ijk_* = the random effect of the nth animal and e*_ij_* = the residual error term. 

Orthogonal polynomial contrasts were used to determine linear and quadratic relationships with increasing INC levels (0, 30, and 60 g/d for each cow). Degrees of freedom were calculated using the Kenward-Roger approximation option of the MIXED procedure. Differences of *p* < 0.05 were considered significant and 0.05 ≤ *p* < 0.10 was considered a tendency.

## 3. Results

### 3.1. Cow Performance and Milk Composition

The analysis of the least squares means for average milk production over the initial 13 weeks post-calving and milk composition during the first 3 weeks is presented in Table 4. The results revealed a significant linear increase in milk production as the levels of INC supplementation escalated (*p* < 0.01). However, no differences were observed in peak milk yield between treatments. The incremental increase in INC levels did not lead to significant variations in the percentage of milk fat, milk protein, or lactose yield. Nevertheless, a linear increase was observed in milk fat yield (*p* < 0.01), milk protein yield (*p* < 0.01), and ECM yield (*p* < 0.01) in response to the increasing levels of INC, accompanied by a linear decrease in somatic cell count (SCC, *p* = 0.04).

The outcomes of our study showed potential reproductive benefits of INC supplementation in dairy cows. We observed a trend towards a reduction in the average number of services required per conception in cows receiving INC (2.60 vs. 1.93 vs. 1.87; *p* = 0.05). No differences were observed in the duration of open days between treatments, although a numerical reduction was observed with the increasing levels of INC (64.53 or 64.67 vs. 73.40; *p* = 0.12).

### 3.2. Blood Parameters

No differences were observed in blood concentrations of NEFA, GLU, TG, or IGF-1 between treatments during any of the studied periods, as detailed in Table 5. Notably, the supplementation of INC resulted in a linear reduction in BHB concentrations throughout the entire study period (*p* = 0.02) as well as during the prepartum period (*p* = 0.02). Conversely, a linear increase in INS concentrations was noted with escalating levels of INC throughout both the entire study period (*p* = 0.01) and the postpartum period (*p* < 0.01). Furthermore, while there was a noticeable rise in INS concentration following calving, there were no significant shifts detected in GLU or IGF-1 levels.

A noteworthy trend in the concentrations of tumor TNF-α and IL-6 indicated a potential decrease with increasing INC levels during the prepartum period (Table 6, *p* = 0.07). No differences were observed in blood concentrations of SOD, GSH-Px, MDA, or T-AOC between treatments during any of the studied periods, as detailed in Table 7. No effects were found on the concentrations of ALT, AST, and TBIL between treatments during any of the monitored periods, as detailed in Table 8. However, the concentration of ALP showed a linear decrease between treatments during the whole period and postpartum, with increasing levels of INC (*p* = 0.03).

### 3.3. Colostrum Compositions

No differences were observed between treatments in colostrum yield or in the concentrations of colostral fat, lactose, colostral protein, IgG, and IgA levels (Table 9). The results revealed a significant linear increase in SOD concentration as the levels of INC supplementation escalated (*p* = 0.02). Additionally, the concentration of POD displayed a linear increase (*p* < 0.01) with escalating INC levels.

### 3.4. Calf Growth Performance and Health

These three groups of calves were fed colostrum produced by the dams of the corresponding treatment group. All calves born in each group were set up as a group, which we still named CON, INC30, and INC60 groups, respectively. Our investigation revealed an absence of calf mortality attributable to severe diarrhea or respiratory illnesses over the duration of the study. No differences in initial BW, BW, ADG, FE, chest girth, or body length were found between the three groups in this study. The wither height of the calves showed a quadratic curve as the INC dose increased (Table 10).

**Figure 1:**
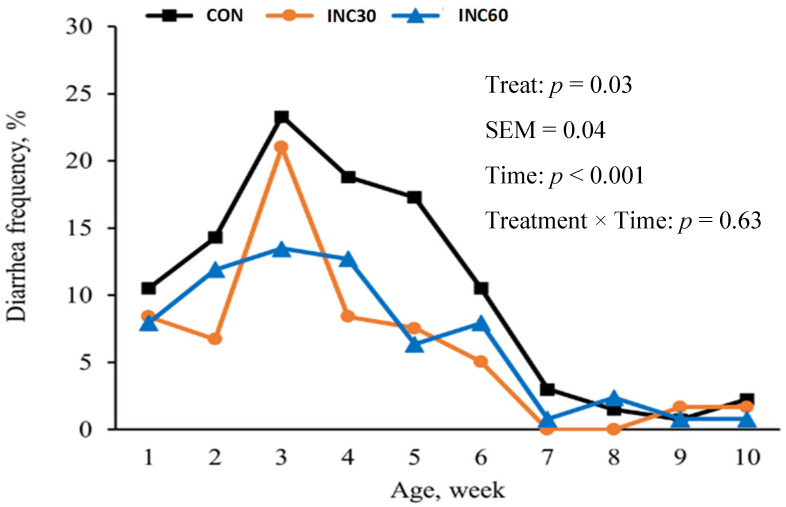
Diarrhea frequency of calves of dams supplemented with 0, 30, or 60 g/d of INC products by week. CON = 0 g/d of INC products (*n* = 15); INC30 = 30 g/d of INC products (*n* = 15); INC60 = 60 g/d of INC products (*n* = 15).

Nevertheless, within the domain of calf health, a substantial disparity in the frequency of diarrhea was conspicuously evident. Specifically, the diarrhea frequency of calves of dams showed a linear decrease as the INC level increased, as illustrated in Figure 1 (10.9% vs. 6.12% vs. 6.08%, *p* = 0.03). Notably, calf rectal body temperature remained consistent and uniform among all treatment groups during each assessment period.

## 4. Discussion

In light of the multifaceted challenges encountered during the transition period in dairy cows, there has been a growing interest in implementing various nutritional strategies to ameliorate the associated physiological and metabolic disruptions [36]. One prominent approach in this regard has been the incorporation of additives possessing immunomodulatory and nutritional regulatory properties. However, the outcomes of previous investigations into the efficacy of such supplementation have exhibited considerable variability with respect to parameters encompassing production performance, hematological indices, and other pertinent facets [37,38]. This variance in findings necessitates a critical imperative for further research endeavors aimed at holistically elucidating the consequences of administering immunomodulatory and nutritional regulatory combined additives throughout the entire transition phase and, concomitantly, deciphering their implications for the subsequent performance of offspring. The present study was meticulously designed to address this knowledge gap and systematically evaluate the repercussions of supplementing dairy cows with an immunomodulatory and nutritional regulatory composite additive during the pivotal transition period. Specifically, the study sought to scrutinize the influence of such supplementation on diverse facets of productive performance and immunological status within the dairy cow population. In addition to these aspects, a comprehensive assessment was conducted to gauge the impact on antioxidant mechanisms, immune responses, and hepatic functions in treated cows. Furthermore, the ramifications of this nutritional intervention on the composition of colostrum were analyzed in tandem with a thorough investigation into the growth trajectory and overall health status of the resultant offspring.

It is noteworthy that our findings align with previous research [39,40,41], because the essential role of B vitamins in critical metabolic processes such as gluconeogenesis, fatty acid synthesis, and protein synthesis in dairy cows is well-established. Furthermore, the components of INC, specifically active dry yeast and Selenomethionine, may have played pivotal roles in the observed improvements in milk production performance [42,43]. However, it is imperative to underscore that further research is indispensable to ascertaining the precise composition and optimal dosages of additives endowed with immunomodulatory and nutritional regulatory functions. This necessity arises from the linear trends observed in our study, implying that there may exist an optimal threshold beyond which supplementation may not yield commensurate gains. Additionally, investigating the intricate mechanisms by which these additives exert their effects, including potential synergistic interactions among their components, would provide a more comprehensive understanding of their utility in dairy cow management during the critical transitional phase. However, the lack of DMI data in our trial is a limitation to the study of overall cow performance, while the focus of this study was on the effects of feeding this product on the immunity of periparturient cows and the growth and health of their offspring, so more systematic trials are needed to study this in the future. Such investigations will not only contribute to optimizing dairy cow nutrition but also hold the potential to enhance the sustainability and productivity of the dairy industry as a whole.

These findings of this study suggest that INC supplementation may enhance breeding efficiency in dairy cows. Our observations find support in a meta-analysis [44], which highlighted that vitamin E supplementation can lead to a decrease in the duration of open days and, when combined with selenium, has the potential to significantly improve breeding efficiency. Vitamin E, a crucial fat-soluble antioxidant, plays a pivotal role in protecting polyunsaturated fatty acids from peroxidation, scavenging free radicals, enhancing biological oxidative stability, and conferring benefits to reproductive organs. Consequently, it can mitigate conception challenges and reduce the likelihood of abortion [45]. Furthermore, the B vitamins, particularly B9 and B12, have been shown to exert beneficial effects on the reproductive system, resulting in a reduced duration of open days [46]. The INC contains both B9 and B12, which may have contributed to the enhanced reproductive performance observed in the INC30 and INC60 groups. During the study period, the occurrence of disease was observed solely within the postpartum phase. A total of nine cases of disease were documented, distributed as follows: two cases within the CON group (1 ketosis, 1 uteritis), four cases within the INC30 group (2 fever, 2 placental retention), and three cases within the INC60 group (2 fever, 1 uteritis), considering a sample size of 15 individuals in each group. The cows that suffered disease in this trial were treated according to the farm’s treatment protocol. Although there were cows in each treatment group that developed disease, it did not have an impact on later production or the trial. To investigate whether there were any noteworthy variations in disease incidence among the treatment groups, a chi-square test was administered as the chosen statistical analysis method. The results of this analysis revealed that there were no statistically significant distinctions detected in terms of disease incidence when comparing the different treatment groups. These findings are consistent with prior research outcomes regarding the application of complex additive supplements in similar contexts [47]. Consequently, the present study’s results align with and substantiate the existing body of research, underscoring the continuity of these observations across multiple investigations.

These observations are in line with previous research endeavors, collectively indicating that BHB concentrations tend to remain unaltered by supplementation [48,49,50]. Thus, it is apparent that while INC supplementation influenced BHB concentrations in our study, the broader hormonal parameters related to energy metabolism exhibited a level of stability that is consistent with prior research in this domain. It is crucial to note that yeast cultures have previously exhibited their capacity to reduce circulating cytokine levels, including IL-6 and TNF-α, in diverse contexts, such as in pigs [51], cellular environments [52], and murine models [53].These observations align closely with the findings of our present study. This intriguing pattern suggests a potential dampening effect on the inflammatory response during the early prepartum phase, consistent with the current observations of reduced TNF-α and IL-6 concentrations with increasing INC levels. Consistently, previous studies have elucidated that liver enzyme activity can exhibit temporal variations during the transition period, and these fluctuations appear to be independent of the physical condition of the subjects, with enzyme activity levels remaining within the normal range [54]. Interestingly, supplementation with 19 g/day of Saccharomyces cerevisiae had no discernible influence on liver function or antioxidative stress capacity during the transition period [55]. In the context of our study, while the evaluated liver function-associated enzymes displayed minimal changes, the notable reduction in ALP concentration could potentially signify a concurrent reduction in hepatocyte damage, suggesting a positive impact on liver activity to some extent. Further investigations are warranted to delve into the intricate molecular mechanisms governing liver function during the critical transition period, aiming for a comprehensive understanding of the underlying dynamics. Intriguingly, no significant effects were observed in the concentrations of T-AOC, GSH-Px, MDA, or SOD in dairy cows across the different treatment groups.

Prior research has also reported that colostral IgG concentrations remain unaffected by the supplementation of yeast cultures or vitamin E in dairy cows [44,56]. However, the concentration and yield of immunoglobulin M (IgM) exhibited quadratic characteristics corresponding to the escalating dosage of INC in this study. The quadratic relationship observed here implies that there may be an optimal level of INC supplementation for maximizing colostral IgM content, which warrants further exploration and consideration in calf health management strategies. Colostral IgM content plays a pivotal role in enhancing immunity and bolstering resistance against bacterial infections in newborn calves [57]. It is essential to recognize that neonatal calves may have inadequate antioxidant defense systems, which can be overwhelmed by the generation of reactive oxygen species [58,59]. Consequently, colostrum with a high antioxidant capacity assumes paramount importance in bolstering calf health and mitigating oxidative stress [60]. Oxidative stress has the potential to induce cellular and DNA damage, rendering calves notably susceptible to gastrointestinal and systemic immune disorders, particularly during the initial fortnight of life. Various enzymatic systems, such as SOD and CAT, present in milk have the potential to ameliorate oxidative stress, potentially enhancing gastrointestinal health in subsequent stages and reducing disease incidence. However, the precise mechanisms underlying their actions warrant further elucidation. These findings highlight the importance of considering the impact of nutritional interventions on antioxidant status, particularly in the context of calf health and early development. In conclusion, while colostrum yield and the concentrations of various colostral components remained largely unaffected by INC supplementation, the intriguing findings related to POD, SOD and IgM content in colostrum offer valuable insights into the potential influence of dietary factors on colostral composition and, consequently, calf immunity and health. These results underscore the complexity of colostrum composition and its responsiveness to dietary interventions, highlighting the need for continued research to optimize colostrum quality for enhanced calf health and productivity.

Supplementing with niacin during the transition period could promote bone development in calves of dams through the nutrient transfer of colostrum [61]. Importantly, our investigation revealed an absence of calf mortality attributable to severe diarrhea or respiratory illnesses over the duration of the study. The well-established association between compromised immunity in both dairy and beef calves and the concomitant rise in occurrences of diarrhea, respiratory ailments, and overall compromised health has been extensively documented [62]. The present study, however, serves as a compelling demonstration of the efficacy of INC supplementation in periparturient cows, effectively reducing the frequency of diarrhea in their calves prior to weaning. This observed benefit may be attributed, at least in part, to the supplemental micronutrient content provided by INC. Calf health is intricately linked with calf immunity, and our comprehensive examination suggests that the evaluated additive has the capacity to mitigate inflammatory responses among calves born to supplemented dams, thereby promoting an improved state of calf health. However, there are still some limitations to our study. It was not clear which specific components of INC positively influenced the reproductive performance and lactation performance of cows in this study and whether it was a single effect of a nutrient element or a combined effect of several nutrients. In addition, further study is necessary to investigate whether the DMI of dairy cows changes after feeding INC and the actual rate of release of the rumen-permeable vitamins of INC into the small intestine. How INC affects calf performance through the dam is also needed to be further investigated.

## 5. Conclusions

In conclusion, INC exhibited benefits in antioxidant stress, anti-inflammatory capacity, and liver function in cows. Further research is warranted to examine how feeding INC affects feed intake and how the components of INC work synergistically to improve immune status and promote performance. Additionally, INC supplementation during the transition period had a positive impact on milk performance metrics, including postpartum milk-fat yield, milk-protein yield, and ECM yield. INC also enhanced colostrum quality without affecting yield. Calves born from cows supplemented with INC experienced significantly reduced preweaning diarrhea, a vital indicator of calf health. Moreover, we found that the optimum level of INC supplementation in this study was 60 g/d by analyzing the results of the performance and biometrics of dairy cows and calves. And these findings suggest that INC offers a promising strategy to enhance both cow and calf health.

## Figures and Tables

**Table 1:** Parity, BCS, and 305-d milk yield of dairy cows at enrollment (d −21 ± 3 relative to expected calving date) and Days of INC feeding.

Variable	Treatment ^1^			SEM	*p*-Value
	CON	INC30	INC60		
*n*	15	15	15		—
Parity	1.93	1.87	1.93	0.80	0.65
BCS	3.43	3.43	3.53	0.15	0.59
305-d milk yield, kg	11,188	11,208	11,275	176	0.75
Days of INC feeding	45.6	44.9	44.9	1.47	0.43

^1^ CON = 0 g/d INC; INC30 = 30 g/d INC; INC60 = 60 g/d INC.

**Table 2:** Ingredients and nutrient composition of cow diets.

Item	Prepartum	Postpartum
Ingredients of basal TMR, % of DM	—	—
Corn silage	61.9	55.7
Steam flaked corn	—	8.40
Cottonseed	—	1.70
DDGS ^1^	2.90	1.40
Soybean hull	—	2.20
Fat powder ^2^	—	1.40
Chopped alfalfa hay	—	6.40
Chopped oat hay	20.1	3.20
Mineral and vitamin premix ^3^	1.70	3.50
Sprayed corn hull	7.20	—
Ground corn	—	5.90
Wheat bran	2.40	2.60
Soybean meal	2.10	6.40
Beet pulp	1.70	1.20
Nutrient composition, % of DM	—	—
CP	15.1	18.0
ADF	28.3	19.3
NDF	45.5	29.8
NFC	30.2	40.4
Starch	16.1	23.6
Crude fat	3.0	4.70
Ash	6.80	8.0
Ca	0.40	1.03
P	0.40	0.40
NEL, Mcal/kg for DM	1.50	1.70

^1^ DDGS: Distillers Dried Grains with Solubles. ^2^ Fat powder: MEGALAC^®^ provided by Yihai Kerry Arawana Holdings Co., Ltd. (Shanghai, China) The main ingredients are C18:1, C16:0, and calcium. ^3^ Prepartum mineral and vitamin premix: 1200 kIU/kg vitamin A, 350,000 IU/kg vitamin D, 18,000 IU/kg vitamin E, 1000 mg/kg Cu, 3000 mg/kg Mn, 4000 mg/kg Zn, 50 mg/kg Se, 100 mg/kg I, 25 mg/kg Co; Postpartum mineral and vitamin premix: 1500 kIU/kg vitamin A, 350,000 IU/kg vitamin D, 20,000 IU/kg vitamin E, 250 mg/kg Cu, 500 mg/kg Mn, 1000 mg/kg Zn, 20 mg/kg Se, 40 mg/kg I, 25 mg/kg Co.

**Table 3:** Chemical composition of calf starter and milk (on DM basis).

Item ^1^	Milk	Starter ^2^
DM	14.3	89.1
CP	3.81	20.3
ADF	—	6.77
NDF	—	19.9
Starch	—	30.5
Crude fat	4.77	2.96
Lactose	4.80	—
Ash	—	7.52

^1^ The nutritive values are the means of the results of the analysis of samples collected each time. ^2^ Provided by Beijing Sunlon Livestock Development Co., Ltd. (Beijing, China) and contained corn, corn bran, corn germ meal, wheat flour, soybean meal, and molasses as the main ingredients.

**Table 4:** The milk yield during the first 13 weeks (d 0–d 90) and milk composition during the first 3 weeks (d 1–d 21) for cows supplemented 0, 30, or 60 g/d INC.

Item	Treatment ^2^			SEM	*p*-Value ^3^		
	CON	INC30	INC60		L	Q	Trt × Time
Milk yield, kg/d	46.8	48.4	49.4	0.95	<0.01	0.61	0.50
Milk composition							
Fat, %	5.46	5.50	5.68	0.20	0.44	0.78	0.65
CP, %	3.35	3.32	3.38	0.05	0.73	0.63	0.46
Lactose, %	5.14	5.28	5.29	0.04	<0.01	0.14	0.94
SCC, ×1000 cells/mL	246	169	166	27.6	0.04	0.28	0.56
MUN, mg/dL	14.9	15.4	16.5	0.56	0.05	0.66	0.98
Yield, kg/d							
Fat	2.14	2.51	2.65	0.11	<0.01	0.43	0.70
CP	1.34	1.50	1.56	0.04	<0.01	0.16	0.03
Lactose	1.61	1.75	1.80	0.04	0.40	0.81	0.31
ECM	49.4	57.5	59.9	1.80	<0.01	0.22	0.43
Peak milk yield, kg	52.3	53.2	54.2	1.35	0.34	0.99	—
DIM at peak milk yield ^1^	46.3	45.9	48.4	6.12	0.81	0.85	—

^1^ DIM = days in milk. ^2^ CON = 0 g/d INC; INC30 = 30 g/d INC; INC60 = 60 g/d INC. ^3^ Orthogonal polynomials were used to test linear (L) and quadratic (Q) effects, Trt × time = *p*-value of the interaction between treatment and time.

**Table 5:** Effects of supplementation with different doses of INC additives on concentrations of serum free fatty acids (NEFA), BHB, triglyceride (TG), insulin (INS), glucose (GLU), insulin growth factor (IGF)-1 during the prepartum (d −21–d 0), postpartum (d 1–d 21) and whole (d −21–d 21) periods.

Item	Treatment ^1^			SEM	*p*-Value ^2^		
	CON	INC30	INC60		L	Q	Trt × Time
BHB, mmol/L							
Whole period	0.43	0.42	0.41	0.01	0.02	0.78	0.09
Prepartum	0.45	0.43	0.41	0.02	0.02	0.90	—
Postpartum	0.42	0.42	0.42	0.01	0.64	0.66	—
NEFA, μmol/L							
Whole period	42.5	43.4	43.2	0.54	0.50	0.54	0.51
Prepartum	41.6	43.8	43.4	1.25	0.17	0.24	—
Postpartum	43.1	43.1	43.1	0.62	0.98	0.98	—
TG, mmol/L							
Whole period	0.21	0.22	0.21	0.01	0.32	0.68	0.36
Prepartum	0.24	0.23	0.22	0.02	0.47	0.95	—
Postpartum	0.20	0.21	0.20	0.01	0.52	0.57	—
GLU, mmol/L							
Whole period	4.18	4.47	4.52	0.17	0.26	0.67	0.62
Prepartum	3.86	4.28	4.02	0.25	0.53	0.13	—
Postpartum	4.39	4.59	4.86	0.23	0.33	0.94	—
INS, mIU/L							
Whole period	23.4	24.7	25.7	0.71	0.01	0.88	0.13
Prepartum	28.1	28.3	27.2	1.04	0.55	0.57	—
Postpartum	20.2	22.2	24.7	0.94	<0.01	0.85	—
IGF-1, ng/mL							
Whole period	120	119	119	1.58	0.80	0.97	0.92
Prepartum	119	119	116	3.26	0.40	0.40	—
Postpartum	120	119	121	2.05	0.78	0.52	—

^1^ CON = 0 g/d INC; INC30 = 30 g/d INC; INC60 = 60 g/d INC. ^2^ Orthogonal polynomials were used to test linear (L) and quadratic (Q) effects; Trt × time = *p*-value of the interaction between treatment and time.

**Table 6:** The effects of supplementation with different doses of additives of INC on concentrations of serum IL-1β, IL-6, serum amyloid A (SAA), haptoglobin (HP), tumor necrosis factor α (TNF-α) and IgG during the prepartum (d −21–d 0), postpartum (d 1–d 21) and whole (d −21–d 21) periods.

Item	Treatment ^1^			SEM	*p*-Value ^2^		
	CON	INC30	INC60		L	Q	Trt × Time
IL-1β, ng/L							
Whole period	52.7	52.1	52.1	0.68	0.60	0.77	0.30
Prepartum	49.6	49.7	50.6	1.56	0.51	0.76	—
Postpartum	54.7	53.7	53.0	0.78	0.25	0.86	—
IL-6, ng/L							
whole period	409	406	399	4.78	0.17	0.82	0.50
Prepartum	422	416	401	11.5	0.07	0.64	—
Postpartum	400	398	398	5.96	0.80	0.93	—
SAA, μg/mL							
Whole period	25.3	25.0	24.6	0.26	0.16	0.85	0.40
Prepartum	26.1	25.7	24.9	0.70	0.11	0.70	—
Postpartum	24.7	24.5	24.4	0.28	0.63	0.92	—
HP, ng/mL							
Whole transition period	49.6	49.1	48.5	0.56	0.24	0.95	0.87
Prepartum	50.8	50.5	48.6	1.46	0.15	0.51	—
Postpartum	48.7	48.4	48.1	0.63	0.74	0.55	—
TNF-α, ng/L							
Whole period	223	212	215	2.96	0.09	0.10	0.57
Prepartum	236	221	219	3.93	0.07	0.37	—
Postpartum	215	207	212	3.42	0.57	0.18	—
IgG, mg/mL							
Whole period	20.8	20.9	20.5	0.35	0.68	0.63	0.92
Prepartum	20.8	21.1	20.2	0.78	0.54	0.38	—
Postpartum	20.8	20.8	20.7	0.45	0.92	0.96	—

^1^ CON = 0 g/d INC; INC30 = 30 g/d INC; INC60 = 60 g/d INC. ^2^ Orthogonal polynomials were used to test linear (L) and quadratic (Q) effects; Trt × time = *p*-value of the interaction between treatment and time.

**Table 7:** Effects of supplementation with different doses of INC additives on concentrations of superoxide dismutase (SOD), glutathione peroxidase (GSH-Px), malondialdehyde (MDA) and total antioxidant capacity (T-AOC) during the prepartum (d −21–d 0), postpartum (d 1–d 21) and whole (d −21–d 21) periods.

Item	Treatment ^1^			SEM	*p*-Value ^2^		
	CON	INC30	INC60		L	Q	Trt × Time
SOD, U/mL							
Whole period	50.8	50.4	51.5	0.53	0.30	0.19	0.91
Prepartum	50.3	50.4	51.8	0.57	0.18	0.48	—
Postpartum	51.1	50.4	51.2	0.60	0.86	0.28	—
GSH-Px, μmol/L							
Whole period	11.1	11.1	10.9	0.23	0.63	0.72	0.33
Prepartum	11.5	11.3	11.2	0.43	0.46	0.47	—
Postpartum	10.7	10.9	10.7	0.31	0.90	0.65	—
MDA, nmol/mL							
Whole period	1.52	1.55	1.50	0.02	0.42	0.12	0.62
Prepartum	1.54	1.54	1.51	0.04	0.54	0.66	—
Postpartum	1.51	1.55	1.49	0.02	0.58	0.10	—
T-AOC, U/mL							
Whole period	8.57	8.66	8.59	0.11	0.33	0.59	0.59
Prepartum	8.64	8.79	8.59	0.21	0.80	0.35	—
Postpartum	8.52	8.57	8.59	0.14	0.73	0.93	—

^1^ CON = 0 g/d INC; INC30 = 30 g/d INC; INC60 = 60 g/d INC. ^2^ Orthogonal polynomials were used to test linear (L) and quadratic (Q) effects; Trt × time = *p*-value of the interaction between treatment and time.

**Table 8:** The effects of supplementation with different doses of additives of INC on the concentration of serum glutamic pyruvic transaminase (ALT), glutamic oxaloacetic transaminase (AST), total bilirubin (TBIL), and alkaline phosphatase (ALP) during the prepartum (d −21–d 0), postpartum (d 1–d 21) and the whole (d −21–d 21) period.

Item	Treatment ^1^			SEM	*p*-Value ^2^		
	CON	INC30	INC60		L	Q	Trt × Time
ALT, U/L							
Whole period	26.6	26.9	24.7	0.86	0.13	0.16	0.62
Prepartum	28.3	28.8	26.7	1.62	0.31	0.37	—
Postpartum	25.5	25.5	23.4	0.79	0.11	0.27	—
AST, U/L							
Whole period	95.1	95.5	94.0	4.19	0.83	0.82	0.63
Prepartum	82.4	81.1	80.8	5.45	0.77	0.91	—
Postpartum	103	105	102	4.34	0.91	0.74	—
TBIL, μmol/L							
Whole period	2.15	2.18	2.16	0.10	0.90	0.85	0.94
Prepartum	1.86	1.79	1.92	0.17	0.72	0.52	—
Postpartum	2.34	2.43	2.32	0.13	0.96	0.55	—
ALP, g/L							
Whole period	41.9	40.1	37.6	1.46	0.03	0.73	0.93
Prepartum	40.5	39.8	38.5	3.59	0.57	0.90	—
Postpartum	42.3	40.3	37.1	1.46	0.03	0.78	—

^1^ Tab1CON = 0 g/d INC; INC30 = 30 g/d INC; INC60 = 60 g/d INC. ^2^ Orthogonal polynomials were used to test linear (L) and quadratic (Q) effects; Trt × time = *p*-value of the interaction between treatment and time.

**Table 9:** The effects of supplementation with different doses of additives in INC on colostrum yield and composition of cows.

Item	Treatment ^1^			SEM	*p*-Value ^2^	
	CON	INC30	INC60		L	Q
Colostrum yield, L	3.67	3.94	3.93	0.48	0.27	0.69
Fat, g/kg	52.1	50.6	53.3	2.14	0.70	0.45
Fat yield, g	191	200	209	14.2	0.43	0.56
Lactose, mg/mL	0.52	0.54	0.51	0.77	0.75	0.52
Lactose yield, mg	1.98	2.12	2.04	0.21	0.56	0.77
Protein, mg/mL	0.77	0.81	0.76	0.04	0.86	0.17
Protein yield, kg	2.83	3.19	2.99	0.28	0.77	0.07
IgG, mg/mL	39.4	39.0	38.0	0.49	0.52	0.88
IgG yield, g	144	154	149	11.2	0.43	0.75
IgA, mg/mL	3.09	3.17	3.13	0.06	0.70	0.30
IgA yield, g	11.3	12.5	12.3	1.01	0.24	0.31
IgM, mg/mL	2.10	2.27	2.15	0.08	0.45	0.05
IgM yield, g	7.71	8.94	8.45	0.61	0.29	0.03
T-AOC, μmol/mL	2.57	2.59	2.66	0.18	0.73	0.84
SOD, U/mL	41.1	42.5	43.2	0.59	0.02	0.75
POD, pg/mL	128	131	132	7.31	<0.01	0.96
GSH-Px, IU/L	421	421	427	16.6	0.87	0.86
MDA, nmol/mL	23.19	22.6	22.75	0.61	0.63	0.61
CAT, U/mL	24.96	24.7	25.42	0.80	0.70	0.58

^1^ CON = 0 g/d INC; INC30 = 30 g/d INC; INC60 = 60 g/d INC. ^2^ Orthogonal polynomials were used to test linear (L) and quadratic (Q) effects.

**Table 10:** The least squares means of starter intake, feed efficiency (FE), and growth performance of calves of dams supplemented with different doses of additives of INC during preweaning (d 1–56), postweaning (d 57–70), and overall (d 1–70) periods.

Item	Treatment ^1^			SEM	*p*-Value ^2^		
	CON	INC30	INC60		L	Q	Trt × Time
Calves, *n*	13	13	13	—	—	—	—
Initial BW, kg	40.1	41.9	40.1	0.93	0.38	0.07	—
Starter intake, g/d							
Preweaning	189	210	222	19.2	0.27	0.37	—
Postweaning	1785	1770	1985	115	0.36	0.55	—
Overall	619	625	685	41.5	0.74	0.75	0.07
BW, kg							
Preweaning	64.7	66.13	64.3	0.74	0.67	0.07	—
Postweaning	102	102	100	1.03	0.45	0.68	—
Overall	70.6	72.1	70.9	0.71	0.76	0.11	0.09
ADG, g/d							
Preweaning	998	970	955	30.90	0.35	0.89	—
Postweaning	910	880	1061	44.40	0.12	0.22	—
Overall	984	954	972	28.30	0.79	0.53	0.58
FE, %							
Preweaning	0.89	0.88	0.84	0.03	0.22	0.76	—
Postweaning	0.52	0.52	0.51	0.03	0.74	0.89	—
Overall	0.81	0.80	0.77	0.03	0.23	0.81	0.96
Withers height, cm							
Preweaning	84.4	85.7	84.2	0.24	0.13	<0.01	—
Postweaning	94.9	95.8	94.9	0.32	0.24	0.04	—
Overall	85.9	87.2	85.2	0.26	0.24	<0.01	0.06
Chest girth, cm							
Preweaning	85.2	87.2	85.9	0.26	0.14	0.02	—
Postweaning	105	106	105	0.44	0.96	0.15	—
Overall	88.8	90.5	89.2	0.21	0.17	0.02	0.25
Body length, cm							
Preweaning	79.7	81.2	79.9	0.34	0.51	0.06	—
Postweaning	93.8	93.8	94.0	0.83	0.47	0.70	—
Overall	81.9	83.1	82.2	0.31	0.63	0.10	0.06

^1^ CON = 0 g/d INC; INC30 = 30 g/d INC; INC60 = 60 g/d INC. ^2^ Orthogonal polynomials were used to test linear (L) and quadratic (Q) effects; Trt × time = *p*-value of the interaction between treatment and time.

## Data Availability

The data presented in this study are available on request from the corresponding author. The data are not publicly available due to relevant data protection laws.
